# Sensitivity to Peer Evaluation and Its Genetic and Environmental Determinants: Findings from a Population-Based Twin Study

**DOI:** 10.1007/s10578-018-0792-x

**Published:** 2018-02-23

**Authors:** Annelie Klippel, Ulrich Reininghaus, Wolfgang Viechtbauer, Jeroen Decoster, Philippe Delespaul, Cathérine Derom, Marc de Hert, Nele Jacobs, Claudia Menne-Lothmann, Bart Rutten, Evert Thiery, Jim van Os, Ruud van Winkel, Inez Myin-Germeys, Marieke Wichers

**Affiliations:** 1 Department of Neuroscience, Research Group Psychiatry, Center for Contextual Psychiatry, KU Leuven, Kapucijnenvoer 35, Box 7001, 3000 Leuven, Belgium; 2 Department of Psychiatry and Neuropsychology, South Limburg Mental Health Research and Teaching Network, EURON, Maastricht University, Maastricht, The Netherlands; 3 Universitair Psychiatrisch Centrum, KU Leuven, Leuven, Belgium; 4 Department of Human Genetics, University Hospital Gasthuisberg, KU Leuven, Leuven, Belgium; 5 Department of Obstetrics and Gynaecology, Ghent University Hospital, Ghent, Belgium; 6 Faculty of Psychology and Educational Sciences, Open University of the Netherlands, Heerlen, The Netherlands; 7 Department of Neurology, Ghent University Hospital, Ghent, Belgium; 8 Department of Psychosis Studies, Institute of Psychiatry, King’s Health Partners, King’s College London, London, UK; 9 Department Psychiatry, Brain Center Rudolf Magnus, Utrecht University Medical Centre, Utrecht, The Netherlands; 10 Department of Neuroscience, Research Group Psychiatry, Center for Clinical Psychiatry, KU Leuven, Leuven, Belgium; 11 Interdisciplinary Center Psychopathology and Emotion Regulation (ICPE), University of Groningen, University Medical Center Groningen (UMCG), University Center Psychiatry (UCP), Groningen, The Netherlands

**Keywords:** Peer evaluation, Gene-environment interactions, Twin design, Bullying, Subjective social status, Adolescents

## Abstract

Adolescents and young adults are highly focused on peer evaluation, but little is known about sources of their differential sensitivity. We examined to what extent sensitivity to peer evaluation is influenced by interacting environmental and genetic factors. A sample of 354 healthy adolescent twin pairs (n = 708) took part in a structured, laboratory task in which they were exposed to peer evaluation. The proportion of the variance in sensitivity to peer evaluation due to genetic and environmental factors was estimated, as was the association with specific *a priori* environmental risk factors. Differences in sensitivity to peer evaluation between adolescents were explained mainly by non-shared environmental influences. The results on shared environmental influences were not conclusive. No impact of latent genetic factors or gene-environment interactions was found. Adolescents with lower self-rated positions on the social ladder or who reported to have been bullied more severely showed significantly stronger responses to peer evaluation. Not genes, but subjective social status and past experience of being bullied seem to impact sensitivity to peer evaluation. This suggests that altered response to peer evaluation is the outcome of cumulative sensitization to social interactions.

## Introduction

Humans have an inherent desire to belong to a group and to be accepted by their peers [1]. Feeling rejected by peers may induce significant stress and may negatively impact psychological, physical, and interpersonal well-being [2–7]. Negative social interactions with peers may threaten the social self in a subtle way, particularly in adolescents and young adults. When compared with children, adolescents show heightened levels of sensitivity and emotional responsiveness to peer evaluation [8]. Elevated sensitivity to peer evaluation during adolescence can in general be considered adaptive, as peer interactions become increasingly salient, and complex social cognitive skills and underlying neural correlates develop [8–12]. However, mental disorders often have their onset during adolescence and early adulthood [13], suggesting that increased sensitivity and reactivity to social interactions may contribute to dysregulation of stress responses and later psychopathology [8, 14, 15].

A considerable amount of peer interactions take place on the internet, with individuals aged 18–25 being the most active group to use social media. Especially adolescents and young adults use social media extensively for their social interactions [16–18]. This may have many advantages, such as being able to connect with people from all over the world and staying in touch with friends on the go [17, 18]. However, it may just as well be harmful for this young age group, since it is rather common to be evaluated and criticized based on an online personal profile. Receiving online evaluations by peers is prevalent among high school and college students and has been found to be at the least as impactful as the real life equivalent [19–21]. Given their frequent exposure to online peer evaluation and its potential detrimental effects on mental health, it is important to study the determinants of sensitivity to peer evaluation in adolescents.

Findings from previous research suggest that exposure to prenatal stress, childhood trauma, and bullying are specific risk factors that may sensitize the individual, contributing to enhanced reactivity to socially stressful events later in life [22–25]. In a study of young adult males, prenatal stress was associated with an altered cortisol response to social-evaluative stress [22]. Experiences of childhood trauma and childhood emotional maltreatment in particular were associated with an increase in sensitivity to social exclusion in a sample of young adults [25]. Also, experiences of bullying have been linked to an altered stress response to social-evaluative stress in adolescents [26] and young adult males [27]. Another *a priori* risk factor for sensitivity to peer evaluation may be a perceived lower social standing within one’s peer group, or ‘subjective social status’. Subjective social status has repeatedly been associated with general and mental health outcomes [28, 29] as well as greater reactivity to social evaluation [30]. This subjective measure of social status captures a broad range of different aspects and weighs income, education, and occupation in proportion to how important the individual finds each aspect in his/her own social context. From an evolutionary perspective, it is plausible that subjective social status may have an impact on sensitivity to evaluation by others. For example, an individual lower in hierarchy may be particularly aware of evaluation by others in order to reduce the risk of exclusion by his/her social group [31].

Previous studies have shown that early environmental exposures may result in ‘behavioral sensitization’ thus contributing to inter-individual differences in sensitivity to social stress, such as peer evaluation [14, 32–34]. Behavioral sensitization refers to a process in which (repeated) exposure to environmental risk factors results in increased biological and behavioral responses to minor stress later in life. Exposure to a range of social adverse experiences early in life may shape later patterns of emotional reactivity, including reactivity to social stressors, such as evaluation by peers. Emotional reactivity to stress has been linked to the development of psychopathology [32, 35, 36]. Individual differences in emotional reactivity to peer evaluation may therefore represent an intermediary phenotype of later psychological symptoms, including psychotic and depressive symptoms [14, 15, 37, 38].

In addition to environmental factors, there is some evidence that an individual’s response to psychosocial stress may be influenced by genetic factors. In particular, there are findings from twin and candidate gene studies that genetic factors may play a role in differences in Hypothalamic–pituitary–adrenal axis (HPA) reactivity to social stressors [39–42]. To date, little attention has been paid to the role of genetic factors on behavioral outcome measures regarding social evaluative stress. Also, environmental and other contextual factors may increase risk in individuals with a susceptible genotype [14, 43–48]. Individuals with a certain genotype may be more susceptible to the effects of, for instance childhood trauma, may respond with dysregulations in HPA axis activity and, in turn, show altered stress reactivity later in life [49]. In other words, it is important not only to examine environmental, contextual and genetic factors in isolation, but also their potential interactions.

The main purpose of this study was to examine the extent to which environmental and genetic factors predict sensitivity to peer evaluation. Using data from a large adolescent and young adult twin study, recruited from a population-based twin register in East-Flanders, Belgium, we aimed to investigate the extent to which environmental and genetic factors, or their interaction, influence sensitivity to evaluation by peers, operationalized as change in negative and positive affect and implicit self-esteem following a structured exposure to online peer evaluation. In this study, we examined the influence of environment as a whole, but also specific environmental risk factors hypothesized to impact stress-sensitization or stress response. These include prenatal stress, childhood trauma, experiences of bullying, and the individual’s subjective position on the social ladder. We tested the following hypotheses: An increase in sensitivity to peer evaluation is associated with (i) environmental risk factors; (ii) genetic factors; (iii) an interaction of genetic and environmental factors.

## Method

### Sample

The study sample consisted of adolescent and young adult twins that were recruited from the East Flanders Prospective Twin Survey (EFPTS). This population-based twin register has prospectively recorded multiple births in the province of East Flanders from 1964 onwards [50]. Zygosity was determined by sequential analysis based on sex, chorion type, umbilical cord blood groups, and in some cases DNA fingerprints. Starting in 2010, individuals of this register between the age of 15 and 34 were invited via a newsletter to take part in a longitudinal study to investigate the role of gene-environment interactions for vulnerability to mental disorders. In order to oversample twins between 15 and 18 years, additional invitational letters were sent to individuals meeting this age criterion. To date, 808 individuals were included in the study. Forty of the individuals were non-twin siblings and 18 were part of a triplet. These individuals were excluded from the current analyses. Of the resulting sample of 750 individuals, 708 took part in the structured peer evaluation task. The project was approved by the local ethics committee and all participants provided written informed consent before study inclusion. For participants under the age of 18 years additional informed consent was obtained from their parents.

### The Digital Social Peer Evaluation Experiment (Digi-SPEE)

**Fig. 1:**
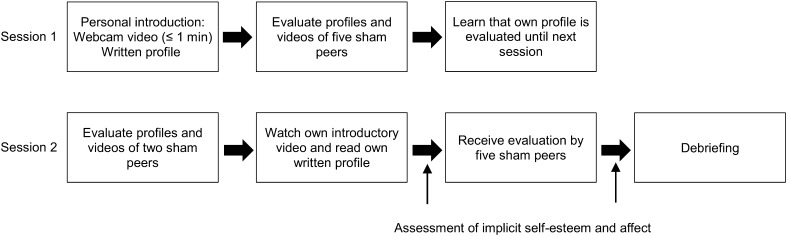
Display of experimental design. The experiment comprised two sessions. During the first session, participants were asked to create a personal profile and rate the profiles of 5 other individuals. A few days later, during the second session, participants had to rate two more profiles, review their own profile, and subsequently received evaluations of five sham participants on their personal profile. Lastly, participants were debriefed about the true nature of the experiment

At its core, communicating online with peers and receiving negative evaluations by peers may be different than receiving it face-to-face [19, 51]. Digi-SPEE is a validated task developed to assess the effects of structured exposure to online peer evaluation similar to what adolescents experience in their daily life (see Fig. 1) [52]. This task was designed to mimic online social network interactions as adolescents and young adults may experience on a regular basis. Peer evaluations as experienced in online social interactions are characterized by a greater level of psychological distance than real-life social encounters, are mostly based on personal traits visible in the individual’s social media profile, and include feedback by their peers. The task was designed to expose participants to subtle negative evaluation of some fundamental personal characteristics (intelligence, stance in life, and appearance).

Participants were told that the general aim of the task was to examine reasons why people like or dislike each other based on online information regarding personal characteristics. During the first session, participants were asked to generate a written profile and short video introducing themselves and to rate five profiles and videos regarding appearance, intelligence, and congeniality using a 7-point scale (higher scores more positive). Participants were led to believe that they rated videos of other participants, when in reality they were presented with videos from five volunteers, which were matched according to age (± 1 year) and gender (three of the same and two of the opposite gender). Participants were then told that peers would evaluate their own profile and video.

The second session took place several days later (mean = 15.5 days; SD = 7.7). Before receiving the evaluation, participants were asked to rate two more profiles, watch their own video and read their own written profile. The evaluation consisted of two filled vertical bars, with one bar stating ‘your evaluation’ and the other ‘average evaluation for all individuals within the study’ (higher fills more positive). For each of the three rated characteristics (intelligence, appearance, and congeniality), the participant’s bar was filled up to approximately halfway, whereas the average bar filled up to approximately 80%. Alongside this general feedback, participants received evaluations (on the three rated characteristics) by five sham participants, of which seven were neutral or positive (e.g., ‘seems friendly’) and eight were mildly negative (e.g., ‘strange nose’).

This structured task consisted of two sessions which were both held in the participants’ homes and were conducted by the same researcher. At the end of the second session, participants were debriefed about the true nature of the task. Menne-Lothmann and colleagues reported in their recently published paper that the majority of participants believed that they were in fact being evaluated by peers [52].

### Design

In order to assess the effects of the Digi-SPEE, a within-subject (pre-post stressor) design was used (see Fig. 1). Implicit self-esteem, positive and negative affect were measured both before and right after the peer evaluation task during the second session [53]. Questionnaires on specific environmental factors were administered at baseline.

### Outcome Measures

Stress sensitivity was operationalized as change in positive and negative affect as well as change in implicit self-esteem from before to after the peer evaluation task.

#### Implicit Self-Esteem

Implicit self-esteem was measured using the Single Category Implicit Association Task (SC-IAT) [54, 55]. Participants were asked to categorize personalized self-words (e.g., their first name) with either positive or negative words. The task comprised two blocks. In the first, self-words have to be sorted in the same category as positive. In the second, self-words have to be sorted into the same category as negative words. The data of this task were prepared in accordance with analysis recommendations from previously published literature on this task [52, 56]. The faster people categorize self-words with positive words relatively to negative words, the higher their implicit self-esteem is (i.e., RT__self+neg. words_−RT__self+pos. words_ in ms). Change scores from before to after the Digi-SPEE were computed (change in implicit self-esteem = implicit self-esteem_after_−implicit self-esteem_before_), so that negative values correspond to a decrease in implicit self-esteem. The psychometric properties of the SC-IAT have been reported by Greenwald and Farnham [54].

#### Positive and Negative Affect

Current positive and negative affect before and after the evaluation were measured using the Positive and Negative Affect Scales (PANAS) [57]. This self-report inventory consists of a positive and a negative affect subscale, each comprising 10 items. For each affect characteristic (e.g., distressed, content, irritated) participants were asked to indicate on a visual analogue scale (VAS) of 105 mm length (with the outer ends labelled ‘not’ and ‘very much’) to what extent they were experiencing this affective state at the moment. Raw ratings (in mm) for positive and negative affect were averaged per person and per assessment. Change scores from before to after the digi-SPEE were computed (change in negative affect = negative affect_after_−negative affect_before_; change in positive affect = positive affect_after_−positive affect_before_), so that negative values correspond to a decrease in positive affect/negative affect. VAS-type instruments have been used widely to assess mood [58–60]. Using a VAS version of the PANAS enabled us to detect small changes from before to after the peer evaluation task. Besides showing higher levels of resolution and thereby providing sensitivity to detect subtle changes, visual analogue scales to assess mood have been shown to be valid and reliable [61]. The internal consistency of PA and NA before the peer evaluation task was alpha = 0.79 and alpha = 0.82, respectively. After the peer evaluation task the internal consistency of PA and NA was alpha = 0.87 and alpha = 0.91, respectively.

### Predictor Measures

#### Prenatal Stress

Birth weight (in gram) was used to estimate prenatal stress [62], with lower birth weight indicating more prenatal stress. Analyses using this estimation for prenatal stress were controlled for gestational age by including gestational age as a predictor to the models [63]. Perinatal data were registered prospectively at birth [64, 65]. Gestational age (number of completed weeks of pregnancy) was based on routine gestational dating, combining last menstrual period and real time ultrasonography in early pregnancy.

#### Childhood Trauma

Childhood trauma was measured using the Dutch shortened version of the Childhood Trauma Questionnaire [66]. This self-report inventory consists of 28 items and covers the following early experiences: emotional, physical, and sexual abuse, as well as emotional and physical neglect. Participants were asked to rate statements such as ‘There was not enough food’ and ‘I was abused’ on a scale ranging from 1 (‘never true’) to 5 (‘very often’). For the analyses a sum score of all subscales was used. The internal consistency of this scale was alpha = 0.89.

#### Experiences of Bullying

An amended questionnaire version of the Retrospective Bullying Interview [48] was used to measure experiences of bullying. This inventory consists of 84 items covering physical, verbal, and indirect forms of bullying during primary school and high school. Furthermore, it contains items measuring the frequency and subjective severity of bullying as well as individual coping strategies. In this study, only the subscales measuring the frequency and subjective severity of bullying were included. The latter subscale consisted of six items measuring the subjective severity of bullying during different life stages on scales ranging from 1 (‘not bullied’) to 5 (‘extremely severe’), giving a maximal score of 30. The Frequency subscale comprised seven items measuring the frequency of bullying during different life stages using scales from 1 (‘not bullied’) to 4 (‘frequently’), giving a maximal score of 28. The internal consistency of the severity and frequency subscales were alpha = 0.80 and alpha = 0.75, respectively.

#### Subjective Social Status

Subjective social status was measured with an amended version of the MacArthur Scale of Subjective Social Status [28]. Participants were presented with an image of a 10-rung ladder with the following description: “See this ladder as a representation of people’s positions in their communities. This may be different for everyone. Choose the community that is of greatest importance to you. At the top of the ladder are the people with the highest position in this community, at the bottom those with the lowest.” Participants were then asked to mark the position on the ladder which best described where they felt they stood relative to other people in their community. This scale has previously been validated in both adolescents [67] and adults [68]. We used a visual analogue scale for this measure, since its degree of resolution offers options of very fine nuance in judgement. Scores could range between 0 and 100 mm. Raw score were used in the current study, with higher scores on this measure indicating higher subjective social status.

### Analyses

First, we estimated the within twin pair similarity of change in implicit self-esteem and affect. Second, the main effects of the specific environmental risk factors on the change scores were examined. Third, we estimated the proportion of variance in implicit self-esteem and affect change scores that was attributable to genetic factors, latent shared environmental factors, and non-shared environmental factors. Last, we investigated whether the impact of adverse environmental factors on sensitivity to peer evaluation is modified by genetic factors. All analyses were carried out using Stata 13.1 (Stata Corporation, College Station, TX, USA) and were controlled for age and gender.

#### Part 1: Within Twin Pair Similarity of Change in Implicit Self-Esteem and Affect

Within twin pair similarity in the outcomes was assessed by estimating intraclass correlation coefficients (ICC) for twin pairs. For each outcome measure, an overall ICC for MZ and DZ twin pairs combined was computed. These ICCs were estimated (based on the ratio of the intercept variance to the sum of the intercept and error variances) using linear mixed-effects models with random intercepts for twin pairs [69].

#### Part 2: Main Effects of Environmental Factors on Change in Implicit Self-Esteem and Affect

The main effects of the specific risk factors on the outcome measures were assessed by adding the specific environmental risk factors as predictors to the aforementioned models. Again, random intercepts for twin pairs were included in these models.

#### Part 3: Latent Genetic and Environmental Influences

First, a specific ICC for MZ pairs and a specific ICC for DZ pairs were computed for each outcome measure. This was done analogous to analyses performed in part 2 using linear mixed-effects models with random intercepts for twin pairs.

Second, using Falconer’s formula [70], the proportion of variance in change in implicit self-esteem, positive affect, and negative affect in response to peer evaluation that is due to genetic factors was estimated. This proportion was defined in terms of heritability *h*^*2*^, where *h*^*2*^ = 2(r_MZ_−r_DZ_), and r_MZ_ and r_DZ_ are the ICCs of a particular outcome for monozygotic (MZ) and dizygotic (DZ) twin pairs, respectively. In addition, the contribution of a shared environment (*c*^*2*^) was estimated by deducting the heritability value from the ICC of MZ twin pairs: *c*^*2*^ = (r_MZ_−*h*^2^). Finally, non-shared environment (*e*^*2*^) is a reflection of the degree to which identical twins raised together are dissimilar and was calculated as follows: *e*^*2*^ = (1−r_MZ_) [71].

To estimate the ICCs, linear mixed-effects models with random intercepts for twin pairs were used once again. Now, the intercept and error variances were allowed to differ for MZ and DZ pairs, so that the ICCs could be computed per zygosity. Models were fitted for each outcome variable (change in implicit self-esteem, positive affect, and negative affect) separately. Wald-type tests were used to examine whether the ICC of DZ pairs differed significantly from the ICC of MZ pairs (in case ICCs do not differ significantly, then this would imply the absence of evidence for a genetic component).

#### Part 4: Associations of Genetic and Specific Risk Factors with Change in Implicit Self-Esteem and Affect

To test the effect of specific environmental risk factors and the latent genetic risk on change in implicit self-esteem and affect, the following four groups were created for each specific risk factor (prenatal stress, childhood trauma, frequency and severity of bullying, and subjective social status): (1) DZ high-score individuals, (2) DZ low-score individuals, (3) MZ high-score individuals, and (4) MZ low-score individuals. The division into high- and low-score groups was done using a median split procedure. Next, we fitted mixed-effects models with random intercepts for twin pairs, but now allowing intercept and error variances to differ for the four different groups mentioned above. Based on these analyses, an ICC for each group was computed. Using these ICCs, *h*^*2*^ was calculated separately for high- and low-scoring individuals. To examine whether the specific environmental risk factors interact with (latent) genetic risk, we tested if *h*^*2*^ of high-scoring individuals differed significantly from *h*^*2*^ of low scoring individuals using Wald-type tests. This was done for each specific environmental risk factor separately.

## Results

### Basic Sample Characteristics

Demographic information for the sample is presented in Table 1. The sample consisted of 708 subjects of whom 256 were MZ and 426 were DZ. The zygosity of 13 twin pairs could not be determined; these were excluded from the genetic analyses (Part 1 and Part 3). The mean age of the participants was 17.8 years (SD = 3.4, range 15–34).

**Table 1:** Characteristics of study population (N = 708)

Age (years), mean (SD, range)	17.8 (3.4,15–34)
Gender, n (%)	
Men	294 (41.5)
Women	414 (58.5)
Gender combination of twin pairs, n (%)	
Same sex female DZ	134 (18.9)
Same sex female MZ	158 (22.3)
Same sex female missing zygosity	14 (2.0)
Same sex male DZ	76 (10.7)
Same sex male MZ	98 (13.8)
Same sex male missing zygosity	12 (1.7)
Opposite-sex	216 (30.5)
Level of education, n (%)	
Elementary school	1 (0.1)
Intermediary vocational education	88 (12.4)
High school	379 (53.5)
Bachelor’s degree	99 (14.0)
Master’s degree	81 (11.4)
Missing	60 (8.5)
Employment status, n (%)	
Homemaker	2 (0.3)
Student	603 (85.2)
Employed	56 (7.9)
Missing	48 (6.8)
Zygosity, n (%)	
MZ	256 (36.2)
DZ	426 (60.2)
Missing	26 (3.7)
Outcome measures, mean (SD)	
Change in implicit self-esteem	− .145 (.451)
Change in positive affect	− 17.9 (40.0)
Change in negative affect	22.0 (48.0)
Predictor measures, mean (SD)	
Birth weight in g	2498 (501.8)
Gestational age in weeks	36.4 (2.0)
Childhood trauma	34.3 (9.0)
Severity of bullying	9.9 (4.4)
Frequency of bullying	10.7 (3.5)
Subjective social status	38.3 (27.9)

*n* indicates the number of individual twins, *MZ* monozygotic, *DZ* dizygotic

#### Part 1: Within Twin Pair Similarity of Change in Implicit Self-Esteem and Affect

Table 2 shows the within twin pair ICCs for all three outcome measures. The correlations suggested that change in implicit self-esteem (ICC = .126; p = .025) as well as change in positive affect (ICC = .111; p = .046) was significantly associated between co-twins.

**Table 2:** Within twin pair intra-class correlation coefficients of outcome measures

	Monozygotic n = 256		Dizygotic n = 426		All n = 682	
	ICC	p	ICC	p	ICC	p
Change in implicit self-esteem^a^	.138	.133	.118	.095	.126	.025
Change in positive affect^b^	.094	.296	.126	.077	.111	.046
Change in negative affect^b^	.113	.222	.026	.714	.058	.306

ICCs of monozygotic and dizygotic twins did not differ significantly from each other

*n* indicates the number of individual twins, Missing values on change scores ^a^n = 63; ^b^n = 52

#### Part 2: Main Effects of Environmental Factors on Change in Implicit Self-Esteem and Affect

Table 3 shows findings on the main effects of the specific environmental risk factors on sensitivity to peer evaluation. Severity of bullying was significantly associated with change in negative affect (b = .787, p = .033): The higher the level of subjective severity of bullying, the larger the increase in negative affect after the task. Individuals with a lower subjective social status showed a stronger decrease in implicit self-esteem (b = .002, p = .022) and in positive affect at trend level (b = .108, p = .066). None of the other risk factors were significantly associated with sensitivity to peer evaluation.

**Table 3:** Analysis of main effects of specific environmental factors on outcome variables (n = 708)

	Change in implicit self-esteem^a^			Change in positive affect^b^			Change in negative affect^b^		
	b	p	95% CI	b	p	95% CI	b	p	95% CI
Birth weight	.000	.995	− .000 to .000	− .002	.581	− .010 to .006	.007	.156	− .004 to .016
Childhood trauma	.002	.440	− .002 to .006	.132	.498	− .249 to .513	− .080	.727	− .526 to .366
Bullying severity	.001	.845	− .007 to .009	− .026	.944	− .736 to .685	.836	.050	− .001 to 1.67
Bullying frequency	.007	.201	− .004 to .017	.210	.657	− .715 to 1.13	.847	.127	− .242 to 1.94
Subjective social status	.002	.016	.000 to .003	.114	.046	− .002 to .227	− .004	.648	− .136 to .128

*n* indicates the number of individual twins, Missing values on change scores ^a^n = 63; ^b^n = 52

#### Part 3: Latent Genetic and Environmental Influences

Table 2 presents specific ICCs for MZ and specific ICCs DZ twin pairs for each outcome measure. Twin pair correlations appeared similar between MZ (change in implicit self-esteem and positive affect: ICC = .138, p = .133 and ICC = .094, p = .296, respectively) and DZ (change in implicit self-esteem and positive affect: ICC = .118, p = .095 and ICC = .126, p = .077, respectively) pairs, suggesting that the observed twin-pair correlations are driven by shared environmental factors rather than genetic influences.

The proportion of variance explained by additive genetic latent factors was not significant for all three outcome measures (respectively; implicit self-esteem, positive affect, and negative affect: 4.0%, p = .863; 0%; p = .789; 17.3%; p = .458). Shared environment explained 9.8% (p = .560) of the variance in change in implicit self-esteem, 15.7% (p = .353) of the variance in change in positive affect, and 0% (p = .723) of the variance in change in negative affect. For all three outcome measures, the largest proportion of variance was accounted for by the non-shared environment component.

All analyses were performed including different as well as same sex DZ twin pairs. However, a sensitivity analysis including only same-sex twins led to the same conclusions.

#### Part 4: Associations of Genetic and Specific Risk Factors with Change in Implicit Self-Esteem and Affect

Table 4 summarizes the results of the analyses examining whether the variation in sensitivity is attributable to the interaction between genetic and specific risk factors. There was no significant association of genetic and specific risk factors with sensitivity to peer evaluation.

**Table 4:** Gene-environment interactions and their association with change in implicit self-esteem, positive affect and negative affect (n = 682)

	Change in implicit self-esteem^A^				Change in positive affect^B^				Change in negative affect^B^			
	ICC	h^2^	p^a^	p^b^	ICC	h^2^	p^a^	p^b^	ICC	h^2^	p^a^	p^b^
Birth weight												
High MZ	.000				.268				.151			
DZ	.154	− .309	*n.e*	*n.e*	.289	− .043	.912	.709	.191	− .079	.875	*n.e*
Low MZ	.270				.097				.171			
DZ	.068	.403	.266		.010	.173	.688		.000	.343	*n.e*	
Childhood trauma												
High MZ	.070				.318			.241	.184			
DZ	.191	− .241	.596	.269	.015	.605	.109		.032	.303	.468	*n.e*
Low MZ	.258				.077				.251			
DZ	.041	.435	.285		.106	− .057	.893		.000	.502	*n.e*	
Bullying severity												
High MZ	.161				.183				.025			
DZ	.147	.029	.951	.803	.067	.234	.570	.676	.067	− .084	.848	*n.e*
Low MZ	.216				.013				.354			
DZ	.126	.180	.634		.017	− .009	.982		.000	.708	*n.e*	
Bullying frequency												
High MZ	*n.e*				.077				.000			
DZ	.119	*n.e*	*n.e*	*n.e*	.048	.057	.909	*n.e*	.163	− .325	*n.e*	*n.e*
Low MZ	.195				.000				.359			
DZ	.182	.023	.951		.213	− .426	*n.e*		.000	.718	*n.e*	
Subjective social status												
High MZ	.339				*n.e*				.000			
DZ	.243	.191	.657		*n.e*	*n.e*	*n.e*		.000	.000	*n.e*	
Low MZ	.000			*n.e*	*n.e*			*n.e*	.069			*n.e*
DZ	.000	.000	*n.e*		*n.e*	*n.e*	*n.e*		.186	− .233	*n.e*	

Missing values on change scores ^A^n=63; ^B^n=52

*n* indicates the number of individual twins. *High* high scores on specific environmental factor. *Low* low scores on specific environmental factor. *MZ* monozygotic, *DZ* dizygotic, *n.e*. could not be estimated due to model conversion problems

^a^Significance of heritability (h^2^)

^b^Significance of differences in h^2^ of two different groups (high, low).

## Discussion

To our knowledge, this study is the first to examine the determinants of sensitivity to peer evaluation in a young general population sample combining a twin design with a novel structured task. We found evidence that people reporting to be more severely bullied in the past were more sensitive to peer evaluation as indicated by a stronger increase in negative affect response following exposure. Furthermore, people who rated themselves as lower on the social ladder showed a stronger decrease in positive affect and implicit self-esteem in reaction to the task. The impact of shared environmental factors remained inconclusive as intraclass correlations suggested an effect but the estimation of variance components did not. We did not find evidence that genetic factors explained a significant proportion of variance in sensitivity to peer evaluation nor that the impact of latent environmental and specific risk factors on sensitivity was modified by genetic factors.

Our findings are in line with earlier work in healthy participants suggesting that bullying [27] and subjective social status [30] impact on sensitivity to evaluative stress as measured with the Trier Social Stress Test (TSST; [53]). However, studies using the TSST also found prenatal stress [22] and childhood trauma [72, 73] to be associated with sensitivity to evaluative stress. This may be due to the nature of the task. In the TSST, people are led to believe that they will be evaluated based on their performance in front of a professional panel, rather than on their personal characteristics by peers as is the case in the digi-SPEE. Based on the current results, we can hypothesize that there may be a certain degree of specificity of the link between quality of prior environmental exposures (i.e., bullying, subjective social status) and the quality of current stressors in the task. Only those specific environmental factors that involved an element of evaluation by peers were associated with an affective response following the digi-SPEE exposure.

There is an extensive body of work on determinants of endocrine and sympathetic responses to experimentally induced evaluative stress. Although the current study focused primarily on affective outcome measures, our findings complement those by Hamilton et al. [27] who reported altered sympathetic responses to evaluative stress in men exposed to bullying. In line with recently published work by Chen et al. [74], we measured *severity* and *frequency* of bullying in order to capture different perspectives of these bullying experiences. While we did find an association between *severity* of bullying and sensitivity to peer evaluation, *frequency* was unrelated to the response to the digi-SPEE. This may be due to the fact that *frequency* of bullying likely also reflects bullying situations that were not serious enough to exert its detrimental effects on individuals. *Severity*, in contrast, reflects a subjective evaluative component and may provide insight into the psychological impact of bullying incidents.

Our findings concerning the association between subjective social status and sensitivity to peer evaluation are in agreement with a recent study conducted by Derry et al. [30]. They found that individuals who see themselves lower on the social ladder show a greater reactivity to brief social-evaluative stress as induced with the TSST. Particularly, the social evaluative component of a stressor may be a crucial aspect in explaining the differences in stress response by individuals with high and low subjective social status [75]. According to earlier work, subjective social status is closely associated with levels of optimism, perceived control, as well as sense of belonging and acceptance [29, 76]. All these aspects may be related to how an individual perceives, and behaves in social interactions with peers. In particular, it may be plausible that through a sense of belonging and acceptance higher subjective social status may create a buffer against social stressors [77].

### The Findings in the Light of Sensitization to Social Stress

The findings of the current study are in line with the hypothesis that social stress sensitivity may be the result of sensitization processes initiated by specific environmental exposures. According to the theory of sensitization, stressors of similar magnitude result in progressively stronger stress responses over time [14, 32, 78]. These processes may finally result in heightened sensitivity to the exposure of social stressors in adulthood, such as negative evaluation by others. It is striking that, in the current study, the two risk factors that are explicitly peer-related in nature (bullying and subjective social status) are the ones that were associated with sensitivity to peer evaluation. With this in mind, we may speculate that the sensitization processes responsible for this effect are specific rather than global in nature. Only previous exposures to *social (peer) evaluative* stressors may have the potential to sensitize people to novel and subtle *social (peer) evaluation* stress encounters, such as those that subjects were exposed to in this structured task. This may also explain why we did not find any associations between other frequently reported environmental stressors, like low birth weight or childhood trauma. However, in the current study, sensitivity to peer evaluation was operationalized in terms of a response in affect and implicit self-esteem. It could very well be that other mechanisms need to be considered in the context of sensitivity to peer evaluation, like threat anticipation, for instance. Further work is needed to provide additional evidence of this specificity for other mechanisms.

### Methodological Considerations

The findings of the current study must be viewed in the light of some methodological considerations. First, the sample of the current study is with a mean age of 17.8 rather young. The findings of our analyses thus cannot be generalized to other age groups. However, as stated above, understanding sources of differences in sensitivity to peer evaluation in this particular group is essential. Individuals of this age show a peak in number of social interactions, as well as elevated levels of sensitivity and emotional responsiveness to these interactions [11, 16, 19].

Second, based on our findings we cannot exclude the possibility that shared environmental factors were associated with the response to peer evaluation. The findings from the ICC analysis provide crude support that there is a certain similarity within twin pairs that cannot be explained by genetic factors and therefore may be related to socio-environmental factors that were shared within pairs which would be in support of a purely socio-environmental pathway to psychopathology [79, 80]. However, this is at variance with contemporary work that suggests several pathways (e.g., environmental and genetic) combine and interact with each other in the development of complaints [81, 82].

We cannot exclude the possibility that unmeasured confounders account for some of the retrospective appraisal, or even bias recall of bullying events and potentially explain some of the variance in the stress response to peer evaluation. Furthermore, it is possible that personality characteristics or other unmeasured factors had an influence on sensitivity to peer evaluation. The role of personality characteristics should be investigated in future research.

The variance components analyses did not suggest any effect of gene-environment interactions on sensitivity to peer evaluation. However, we cannot exclude the possibility that gene-environmental interactions do play an important role. As unaccounted gene-environment interactions may be summarized in the non-shared environment component [83], and the biggest proportion of variance in sensitivity to peer evaluation was explained by this component, it may be that gene-environment interactions or epigenetic processes [84] are involved in the development of sensitivity to peer evaluation. Nonetheless, our analyses on specific risk factors did not show evidence for gene-environment interactions. This may be due to the complexity of the models employed as well as the use of binary risk factors, which may imply a reduction in power (however, in the context of the models we fitted, there is no straight forward method of letting h^2^ be a function of a continuous covariate).

The current study did not investigate timing effects of bullying, as to what extent experiences during primary school are differently associated with sensitivity to peer evaluation than more recent events during secondary school. It has been suggested by recent studies that bullying experiences during both primary and secondary school are associated with alterations in health and mental health [85–87]. However, the phases of primary and secondary school may each be marked by different critical developmental processes [88, 89], and therefore experiences of bullying may have different *consequences* for the personal development. Future studies should therefore investigate further, whether experiences of bullying during these distinct developmental stages have a differential impact on sensitivity to peer evaluation.

Finally, our bullying measure focused on aspects of ‘traditional’ bullying rather than ‘cyberbullying’. Although similar in various ways, recent findings suggest that these two forms of bullying are two distinct concepts [90, 91]. In this era of increased social media use and internet communication—especially among adolescents and young adults [16–18]—it would therefore be worthwhile to study the differential effects of (early) cyberbullying experiences on online peer evaluation in future research.

## Conclusions

In conclusion, we found support for the hypothesis that sensitivity to peer evaluation may be the result of specific sensitization processes initiated by adverse experiences. The findings of the current study provide first evidence that subjective social status as well as bullying are among potential risks associated with sensitivity to peer evaluation. Evidence on the determinants of differential sensitivity to peer evaluation in such a young sample of adolescents and young adults is vital for gaining a better understanding of when and how to intervene best. Assessment and monitoring of subjective social status provides useful information on future health risk [92]. Interventions targeting feelings of social belonging may decrease this risk, as individuals that feel confident in their belonging may experience the social world in a way that it may be self-reinforcing. As a consequence, they may initiate more relationships and obtain opportunities for growth and belonging, which in turn promote well-being [93].

## Summary

Peer interactions increasingly take place on the internet. Especially adolescents and young adults use social media extensively for their social interactions [16, 94]. The use of social media may have many advantages, such as being able to connect with people from all over the world and staying in touch with friends on the go. However, they may just as well be harmful for this young age group, since it is rather common to be evaluated and criticized based on an online personal profile. Receiving online evaluations by peers, has been found to be at the least as impactful as the real life equivalent. We examined in a sample of 354 healthy adolescent twin pairs (n = 708) to what extent sensitivity to peer evaluation is influenced by interacting environmental and genetic factors. They took part in a novel structured task in which they were exposed to peer evaluation comparable to online social interactions. Sensitivity to peer evaluation was operationalized as changes in affect and implicit self-esteem from before to after exposure. The proportion of the variance in sensitivity to peer evaluation due to genetic and environmental factors was estimated, as was the association with specific *a priori* environmental risk factors. Differences in sensitivity to peer evaluation between adolescents were explained mainly by non-shared environmental influences. No impact of latent genetic factors or gene-environment interactions was found. Adolescents with lower self-rated positions on the social ladder or who reported to have been bullied more severely showed significantly stronger responses to peer evaluation. Taken together, not genes, but subjective social status and past experience of being bullied seem to impact sensitivity to peer evaluation. This suggests that altered response to peer evaluation is the outcome of cumulative sensitization to social interactions.
